# Reply to Letter to the Editor: Regarding “The Role of Obesity in Pediatric Orthopedics”

**DOI:** 10.5435/JAAOSGlobal-D-20-00012

**Published:** 2020-02-20

**Authors:** Philip Nowicki, John Kemppainen, Lisa Maskill, Jeffrey Cassidy

**Affiliations:** Grand Rapids, MI

*The Authors' Reply:* Thank you for your letter regarding our article on the role of obesity in pediatric orthopedics. You raised multiple concerns and disagreements regarding our conclusions on the effects of obesity on the growing skeleton and in pediatric trauma.^1^ We are sorry to hear that you found some of our statements misleading or inaccurate, and we will address these concerns one by one to provide clarification.

It was asserted that the review articles by Bialo and Gordon^2^ and Lazar-Antman and Leet^3^ did not support our comment that obesity leads to an overall lower bone mass when compared with overall patient weight in children. These articles, review articles in themselves, cited other articles that made these conclusions. In the article by Bialo and Gordon,^2^ they acknowledge that “the definitive effect of pediatric obesity on bone mass…remains controversial,” but later on in their article (in the same article section), they cite an article by Wetzsteon et al^4^ that found that the increased bone strength in obese children remained disproportionate to their overall bony mass and may possibly lead to an increase risk of fracture.^2^ Looking closely at their data, Wetzsteon et al^4^ mention in their discussion that although multiple studies have reported higher bone mineral density and bone mineral content in overweight/obese children, this idea requires refining in light of their results which showed that although absolute bone strength is higher in overweight children and adequate for higher muscle mass, the overall strength is low relative to the larger fat mass and higher body weight in overweight children.^4^ They hypothesized that although there is an overall increased bone strength in overweight children leading to larger load bearing with regular locomotion, these bones are not adapted for unusual loading as in the case of a fall leading to higher bone strain rates which may lead to an increase fracture risk.^4^ Regarding the article by Lazar-Antman and Leet,^3^ their review on the subject found the available data on lower bone mass for weight in obese children unclear but did not definitively assert that to be untrue as they discussed multiple studies that found overall bone content in obese children to be lower than their overall size or predicted value for size. Considering this, they did not recommend routine bone mineral density assessment in obese children who had not sustained a fracture to predict future fracture risk.^3^ Therefore, to say that our conclusions are not supported by these articles is not complete. There continues to be knowledge gaps on exactly how much influence obesity alone has on the growing skeleton, and the impetus for our article from the beginning was to see exactly where the literature lay on the subject. There is room for improvement in the pediatric orthopaedic literature.

The article cited by Li et al^5^ evaluated a large inpatient database (KID database) and found that obese children were 16 times less likely to sustain a fracture than their normal weight counterparts. We evaluated similar studies from large inpatient databases which have inherent flaws as they attempt to glean generalized information from codes entered into a computer rather than on prospective data and therefore must rely on accurate data input from coders and other nonphysicians. Conclusions from these study types should be taken carefully, as they offer an assessment of an overall pattern for a particular question rather than provide firm conclusions on true causality or risk. This concern was brought up in the peer review for this article and addressed to the best of our ability with the available data at hand as true prospective studies on which injury patterns will occur at the time of trauma are impossible to perform, leaving researchers with no other choice than to assess hospital database information. In addition, although Li et al^5^ found obese children were less likely to sustain fractures than normal weight patients, they had a higher rate of open reduction and internal fixation for similar fractures along with greater inpatient complications. This conclusion pars with our discussion on how obesity can lead to greater severity in fractures relative to healthy weight children and that obesity plays a role in affecting pediatric orthopaedic management. This article was also published in April of 2019, one month before our article, and thus would not have been available for reference; we are grateful for bringing it to our attention as it offers an apt addition to our article.

The article by Seeley et al^6^ was cited and stated that the authors did not review the radiographs of their subjects in the article results. We find this to be inaccurate. Although the radiographs themselves were not discussed in the article's results (ie, Baumann angles, humeral-capitellar angle, etc), this does not imply the authors did not assess the radiographs of their patients. There is no way to classify a fracture by any radiographic classification system without assessing radiographs at some point in the treatment of an individual patient. Seeley et al^6^ provide a thorough review of their fracture patterns as it was the point of their article. They may not have commented on them for the article itself, but they did classify their fractures at least remotely at the time of injury, reflecting the retrospective nature of their data. A reference was made to a comment in our article on the influence of obesity on early epiphyseal plate closure, which came from the caption of Figure 3. This was not directly or solely linked to the article by Seeley et al^6^ but from a discussion found earlier in our article on the section reviewing the effect of obesity on the growing skeleton. There we reference the article by Shalitin and Kiess^7^ who asserted that “obese children are frequently tall for their age, with accelerated epiphyseal plate maturation despite low growth hormone levels.” We do not find this misleading as you say because we support the conclusion with evidence found and reviewed by previous authors. The figure was placed in this section as an example of how obesity can lead to greater fracture complexity compared with healthy weight children.

We again appreciate the comments regarding our article “The Role of Obesity in Pediatric Orthopedics.”^8^ We stand by our conclusions based on the available literature and believe our article provides special interest and guidance to the reader, providing recommendations for orthopaedic surgeons to help improve the care given to overweight and obese children in both nonsurgical and surgical care settings.
